# Palmitate acid promotes gastric cancer metastasis via FABP5/SP1/UCA1 pathway

**DOI:** 10.1186/s12935-019-0787-0

**Published:** 2019-03-22

**Authors:** Jiaomeng Pan, Qingqiang Dai, Tianqi Zhang, Chen Li

**Affiliations:** Department of Surgery, Shanghai Key Laboratory of Gastric Neoplasms, Shanghai Institute of Digestive Surgery, Ruijin Hospital, Shanghai Jiao Tong University School of Medicine, Shanghai, 200025 People’s Republic of China

**Keywords:** Palmitate acid, Gastric cancer, SP1, FABP5, Metastasis, UCA1

## Abstract

**Background:**

Gastric cancer (GC) has a clear predilection for metastasis toward omentum which is primarily composed of adipose tissue, combine with our previous research that long non-coding RNA Urothelial cancer associated 1 (UCA1) could promote the peritoneal metastasis of GC, we put forward the hypothesis that fatty acids (FAs) might contribute to these phenomena and a connection between FAs and UCA1 might exist.

**Methods:**

TCGA database was applied to investigate the expression levels of UCA1 in GC tissues and normal gastric tissues and its correlation with GC patients’ survival. Transfection of siRNA was utilized to knockdown cellular levels of FA-binding protein 5 (FABP5), SP1, UCA1. Migration assay and invasion assay were performed to assess the biological effects of palmitate acid (PA), FABP5, SP1 and UCA1 on GC metastasis. The underlying mechanism was investigated via western blot, immunofluorescence (IF), semi-quantitative RT-PCR (sqRT-PCR) and quantitative RT-PCR (qRT-PCR) analysis.

**Results:**

Here we demonstrated that PA could promote the nuclear transport of FABP5, which then increased the nuclear protein levels of SP1. Consequently, GC cellular expression levels of UCA1 were increased which promoted the metastatic properties of GC. Besides, the cellular levels of UCA1 in GC tumor tissues were significantly higher than that in normal tissues. Its levels in GC tumor tissues also negatively correlated with the prognosis of GC patients using TCGA database.

**Conclusions:**

Our research revealed the potential tumor-promoting effect of FA transport protein FABP5. We also established a connection between non-coding RNA and FA metabolism, treatment targeted either to patients’ diets or FABP5 might improve the prognosis of GC patients.

## Background

Gastric cancer (GC) stands as one of the most commonly diagnosed cancers and the third leading cause of cancer-induced death worldwide which has induced tremendous burdens throughout the world especially in eastern Asia [1–3]. High rate distal metastasis is one of the major factors influencing the prognosis of GC patients [3–5]. Therefore, investigating the underling mechanisms contributing to the metastasis of GC is urgent to improve the prognosis of GC patients.

Fatty acid (FA) metabolism has recently been reported to contribute to the initiation and progression of multiple types of cancers [6–8]. GC has a clear predilection for metastasis toward omentum primarily composed of adipose tissues [9]. Considering the vital role of microenvironment in tumor progression, we put forward the hypothesis that FAs may promote the GC metastatic cascade. FAs include saturated FAs, monounsaturated FAs and polyunsaturated FA. Li [10] found monounsaturated fatty acid oleic acid (OA) could be consumed by highly metastatic carcinoma cells to maintain malignancy in an AMPK-dependent manner while it inhibited cancer cell growth and survival in low metastatic carcinoma cells. Several researchers have also demonstrated polyunsaturated FAs suppressed GC progression [11, 12]. However, the biological effects of saturated FAs on GC have not been demonstrated.

FA binding proteins (FABPs) are involved in FAs transport and metabolism. FABP5 has been reported to participate in the development of multiple cancers [13–15], however its biological effect on GC has not been explored. Recent studies illustrated that, upon association with particular ligands, FABP5 translocated from the cytosol to the nucleus and then directly performed its biological effects within nucleus [16]. Despite multiple ligands of FABP5, only particular compounds are able to trigger its nuclear translocation [16]. Di-Poi demonstrated that activation of PPARβ/δ could significantly repress the expression level of PTEN [17], indicating FABP5 might participate in the tumor progress via mediating its FA metabolism.

Long non-coding RNA Urothelial cancer associated 1 (UCA1) has recently been reported to participate in multiple tumor progression [18–21]. We previously demonstrated UCA1 improved GC metastasis by regulating GRK2 protein stability via promoting Cbl-c-mediated GRK2 ubiquitination and degradation, which then activated the ERK-MMP9 pathway. Combined with the fact that GC has a clear predilection for metastasis toward omentum, we put forward the hypothesis that a connection between FAs and UCA1 might exist.

In this research, we demonstrated that PA could activate FABP5 and promote its nuclear transport, which then increased the nuclear levels of SP1. Consequently, cellular levels of UCA1 were increased and both the migratory and invasive properties of GC were improved.

## Methods

### Cell lines and reagents

GC cell lines HGC27 and MGC803 were purchased from Shanghai Institutes for Biological Sciences, Chinese Academy of Sciences. All cell lines were grown in RPMI-1640 medium (Gibco, BRL, San Francisco, CA, USA) supplemented with 5 μg/ml penicillin/streptomycin and 10% fetal bovine serum (Invitrogen, Carlsbad, CA, USA) in a humidified incubator at 37 °C with 5% CO_2_.

PA (Cat#P9767, Sigma) was prepared as a 2 mM stock solution by dissolving it in ddH_2_0 at 70 °C and filtered at 0.22 μm and stored at 4 °C. The stock solution was then added into 2% FA-free Bovine Serum Albumin (BSA) medium made by dissolving 2% w/v BSA (Cat#A7030, Sigma) in serum-free RPMI-1640 medium (Gibco, BRL, San Francisco, CA, USA) supplemented with 5 μg/ml penicillin/streptomycin to achieve the desired final concentration.

### TCGA database analysis

GC gene expression data (mRNA, normalized RNAseqV2 RSEM) was retrieved from TCGA database. Receiver operating characteristic (ROC) was plotted and an optimal cut-off value was applied to classify patients into low- and high-expression groups referred to the method elucidated by Xiang [22] according to the expression level of UCA1. The survival rate of these two groups was then calculated by Kaplan–Meier method and log-rank test using GraphPad Prism 6.0 (Inc., La Jolla, CA, United States).

### Semi-quantitative RT-PCR (sqRT-PCR) and quantitative RT-PCR (qRT-PCR)

Total RNA was extracted using TRIzol reagent (Invitrogen) and cDNA synthesis was performed using a reverse transcription kit (Promega, Madison, WI, USA) according to the manufacturer’s instructions. For sqRT-PCR, PCR was performed on the cDNA using the relate primers. The products from the PCR were run on a 1% agarose gel containing ethidum bromide and visualized under a UV light. For qRT-PCR, the mRNA level of UCA1 was measured using the SYBR Green PCR Master Mix (Applied Biosystems, Waltham, MA, USA) and the Applied Biosystems 7900HT sequence detection system (Applied Biosystems). UCA1 mRNA relative expression level was evaluated using the 2^−ΔΔCt^ method and normalized to glyceraldehyde 3-phosphate dehydrogenase (GAPDH).

Primers sequences were listed in Table 1.

**Table 1:** Sequences of siRNAs and primers

Names	Sequence (5′–3′)
si-FABP5#1	AGGAGUUAAUUAAGAGAAUGA
si-FABP5#2	UGAAUACAUGAAGGAGCUAtt
si-FABP5#3	GUCACCUGUACUCGGAUCUAU
si-SP1	Forward: CCAACAGAUUAUCACAAAU
	Reverse: UUGUUUGUCUGAUCGGCUCTT
si-UCA1	Forward: GAGCCGA UCAGACAAACAATT
	Reverse: UUGUUUGUCUGAUCGGCUCTT
Primers: GAPDH	Forward: GGACCTGACCTGCCGTCTAG
	Reverse: GTAGCCCAGGATGCCCTTGA
Primers: UCA1	Forward: CTCTCCATTGGGTTCACCATTC
	Reverse: GCGGCAGGTCTTAAGAGATGAG

### RNA interference

A small interfering RNA (siRNA) that specifically target SP1 and its negative control were kindly granted by Wang [19, 20] with sequences listed in Table 1. A small interfering RNA (siRNA) specifically targeting human lncRNA UCA1 and its negative control were also kindly granted by Wang [19, 20] with sequences listed in Table 1. FABP5 siRNA sequences and negative control sequences were designed and synthesized by Genomeditech (Genomeditech, Shanghai, China) listed in Table 1. MGC803 and HGC27 cells were cultured in six-well plates and transfected with siRNA using Lipofectamine 2000 reagent (Invitrogen) following the manufacturer’s protocol. Knockdown of UCA1 was confirmed by qRT-PCR and sqRT-PCR, knockdown of FABP5 and SP1 were confirmed by western blot analysis.

### Western blot analysis

Cell samples were prepared in RIPA cell lysis buffer (Solarbio, Beijing, China) containing protease inhibitor phenylmethane sulfonyl fluoride. The concentration of protein was quantified using a bicinchoninic acid protein assay kit (Pierce, Rockford, IL, USA) based on a bovine serum albumin standard curve. A total of 25 μg protein was loaded onto a 10% sodium dodecyl sulfate polyacrylamide gel and then transferred onto 0.22 μm PVDF membranes (Millipore, MA, USA). The membranes were blocked with 1 × TBST buffer containing 5% bovine serum albumin and incubated with the corresponding primary antibodies at 4 °C overnight. After the addition of secondary antibodies, membranes were visualized with Thermo Pierce chemiluminescent (ECL) Western Blotting Substrate (Thermo, Waltham, MA, USA) using a Tanon 5200 system (Tanon, Shanghai, China). The antibodies involved were purchased from Cell Signaling Technology.

### Cell migration and invasion assays

Cell migration and invasion assays were performed using 24-well plates and 8 μm transwell inserts (Corning Life Science, Acton, MA, USA). For migration assays, tumor cells were suspended in 200 μl serum-free RPMI-1640 medium (4 × 10^4^ cells) containing either 2% BSA or 0.1 mM PA and cultured in the upper chamber. Fetal bovine serum-conditioned medium (10%) (700 μl) was added to the lower 24-well plates. For invasion assays, the inserts were coated with Matrigel (50 μl/well) (BD Biosciences) and kept in a humidified incubator at 37 °C with 5% CO_2_ for at least 2 h before adding the cells, tumor cells were suspended in 200 μl serum-free RPMI-1640 medium (1 × 10^5^ cells) containing either 2% BSA or 0.1 mM PA and cultured in the upper chamber. After a period of culture, tumor cells remaining in the upper side of the inserts were removed with cotton swabs. Tumor cells that migrated to the lower side of the inserts were fixed in methanol and stained with 0.5% crystal violet for 30 min at room temperature. Migrated cells were photographed using Nikon Digital Sight DS-U2 (Nikon, Tokyo, Japan) and Olympus BX50 microscopes (Olympus Optical Co. Ltd., Tokyo, Japan). Five visual fields were randomly chosen to calculate the number of migrated cells.

### Immunofluorescence (IF)

Cell samples were fixed in 4% paraformaldehyde at room temperature for 60 min. Sections were gently washed three times in 1 × phosphate buffered saline (PBS) and then permeabilized with 0.25% Triton (Cat#T9284, Sigma) in 1 × PBS for 10 min. After being blocked with 3% bovine serum albumin for 1 h at room temperature, the sections were incubated with primary antibodies for 1 h at room temperature and gently washed three times in 1 × PBS. Sample sections were then incubated with Alexa Fluor 488 dye-conjugated secondary antibody and DAPI (Cat#4083S, CST). Pictures were taken using an Olympus BX50 microscope (Olympus, Tokyo, Japan).

### Statistical analysis

All experimental results were repeated at least three times and are shown as mean ± standard deviation (s.d.). Differences between treated and control groups were analyzed using the Student’s t-test and one-way ANOVA. A two-tailed value of P < 0.05 was considered statistically significant. All statistical analyses were performed with the Stata software 12.0 (Stata Corporation, College Station, TX, USA).

## Results

### UCA1, upregulated by PA, overexpresses in GC tissues and correlates with poor survival

**Fig. 1:**
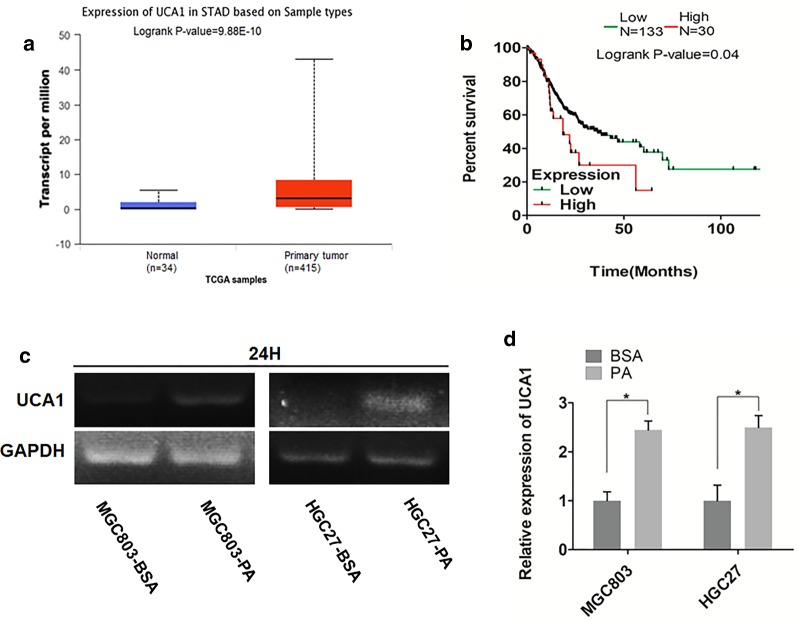
UCA1, up-regulated by PA, overexpresses in GC tissues and correlates with poor survival. **a** Analysis of UCA1 expression levels between normal and primary tumor samples in GC using TCGA database. **b** The association of expression level of UCA1 with GC patients’ survival. **c**, **d** sqRT-PCR and qRT-PCR analysis of cellular levels of UCA1 in GC cell lines MGC803 and HGC27 treated with either 2% BSA or 0.1 mM PA for 24 h. Data are shown as mean ± SD of three independent experiments. *P < 0.05, **P < 0.01, ***P < 0.001

To validate the biological functions of UCA1 in GC progression, we investigated the expression levels of UCA1 in GC tissues using TCGA database and found UCA1 significantly overexpressed in GC tissues compared to normal gastric tissues (Fig. 1a). Besides, the expression levels of UCA1 in GC tissues significantly correlated with the poor survival of GC patients (P = 0.04, Fig. 1b), indicating UCA1 might participate in the development of GC.

Considering that UCA1 contributed to GC metastasis toward omentum which was primarily composed of adipose tissues, we then explored the relation between PA and UCA1. We incubated GC cell lines MGC803 and HGC27 with either 2% BSA or 0.1 mM PA for 24 h and found the cellular levels of UCA1 were significantly elevated by PA compared to its control groups detected by either sqRT-PCR (Fig. 1c) or qRT-PCR (Fig. 1d). Taken together, PA might promote GC migration and invasion through increasing the cellular levels of UCA1.

### PA promotes GC cell migration and invasion through UCA1

**Fig. 2:**
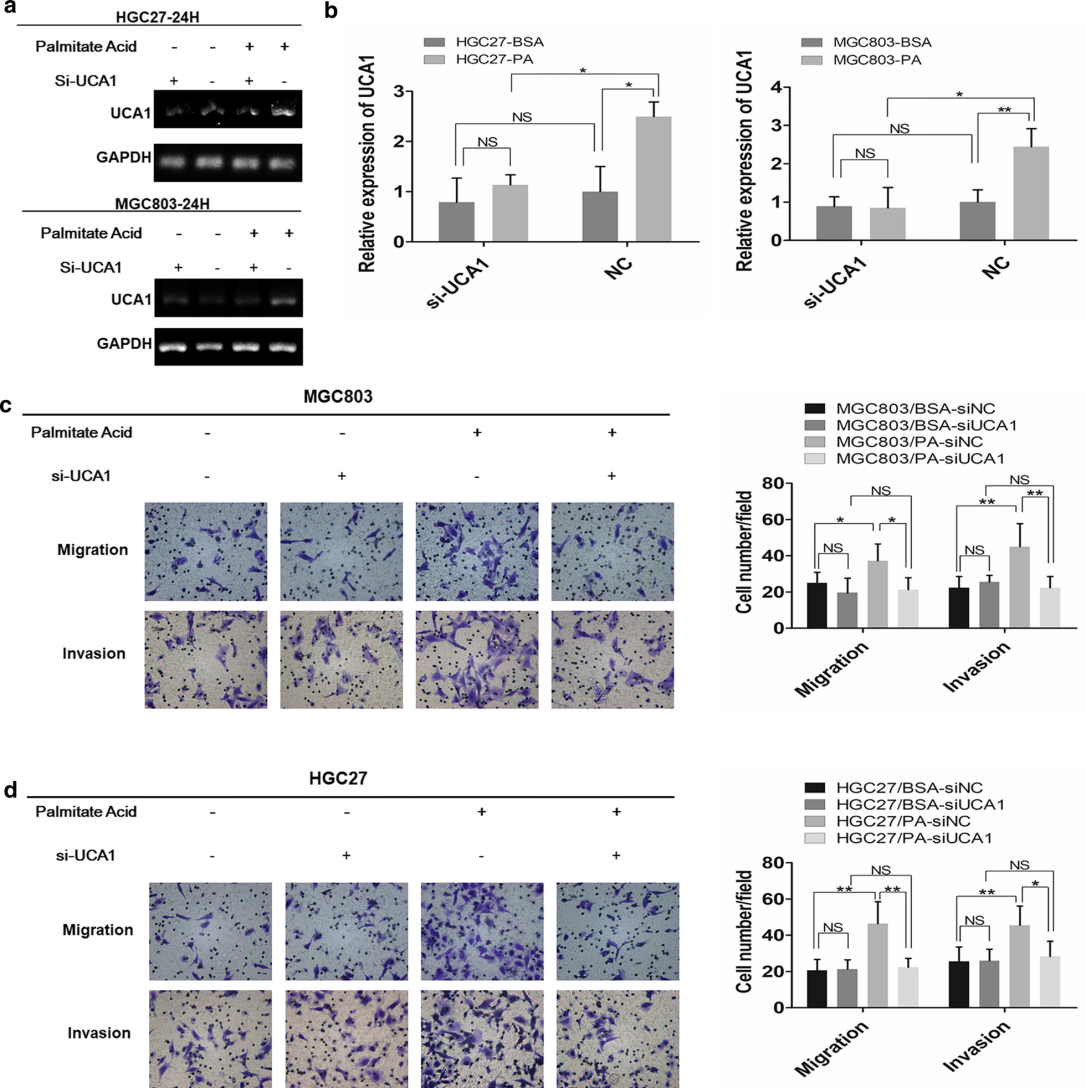
PA promotes GC cell migration and invasion via UCA1. **a**, **b** sqRT-PCR and qRT-PCR analysis of cellular levels of UCA1 in MGC803 and HGC27 transfected with siRNA of UCA1 or its negative control and then treated with either 2% BSA or 0.1 mM PA for 24 h respectively. **c**, **d** The effects of either 2% BSA or 0.1 mM PA on GC cell migration and invasion transfected with either siRNA of UCA1 or its negative control (magnification: ×200). Histograms show the number of migrated and invaded cells (magnification: ×200). Five random fields are selected for statistical analysis. Data are shown as mean ± SD of three independent experiments. *P < 0.05, **P < 0.01, ***P < 0.001, ‘NS’ means not significant

To testify whether PA could promote GC metastasis via UCA1, we specifically knockdown cellular UCA1 using siRNA and then performed migration and invasion assays on MGC803 and HGC27 treated with either 2% BSA or 0.1 mM PA. The efficiency of si-UCA1 was validated by either sqRT-PCR (Fig. 2a) or qRT-PCR (Fig. 2b). We discovered that 0.1 mM PA could significantly promote both the migratory and invasive properties of GC cell lines compared to their control groups which could be inhibited by knockdown of UCA1 (Fig. 2c, d). This result was not found between BSA treated groups (Fig. 2c, d), indicating that PA could indeed improve the GC metastatic abilities through elevating the cellular levels of UCA1.

### PA elevates nuclear protein level of SP1 to enhance UCA1 expression level and promote GC metastasis

**Fig. 3:**
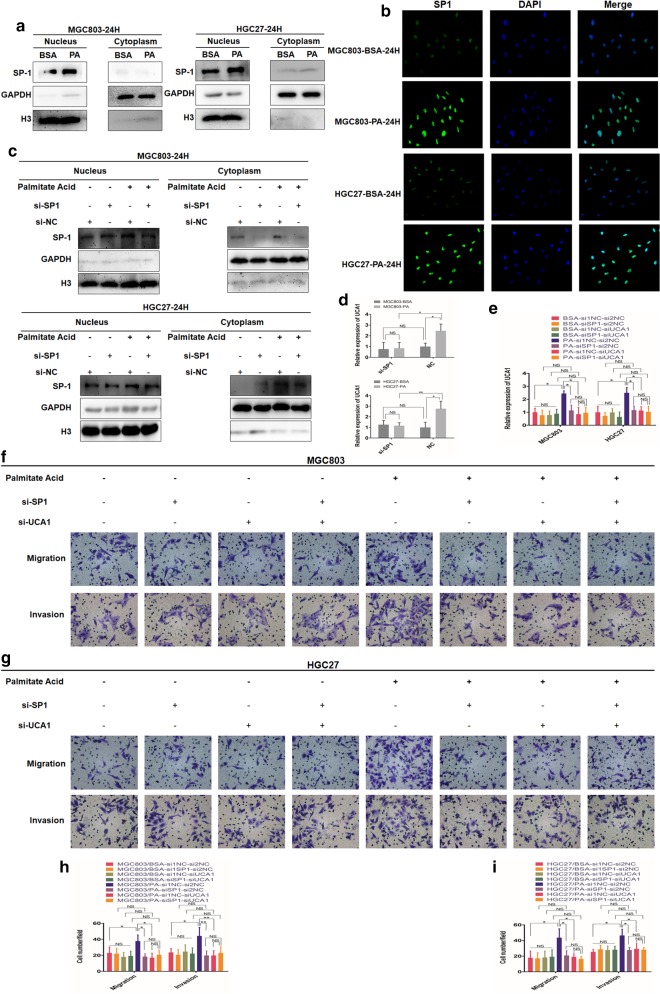
PA enhances GC nuclear protein levels of SP1 to elevate cellular UCA1 and promote GC metastasis. **a** Western blot analysis of nuclear and plasm protein levels of SP1 in MGC803 and HGC27 treated with either 2% BSA or 0.1 mM PA. **b** IF images represent cellular protein levels of SP1 and its distribution of MGC803 and HGC27 treated with either 2% BSA or 0.1 mM PA. **c** Western blot analysis of the efficiency of siRNA of SP1. **d**, **e** qRT-PCR analysis of cellular levels of UCA1 in MGC803 and HGC27 transfected with si-SP1, si-UCA1 or both and their negative controls respectively and then treated with either 2% BSA or 0.1 mM PA for 24 h. **f**, **g** The effects of transfection with si-SP1, si-UCA1 or both and their negative controls on GC cell migration and invasion treated with either 2% BSA or 0.1 mM PA (magnification: ×200). **h**, **i** Histograms show the number of migrated and invaded cells (magnification: ×200). Five random fields are selected for statistical analysis. Data are shown as mean ± SD of three independent experiments. *P < 0.05, **P < 0.01, ***P < 0.001, ‘NS’ means not significant

Based on our previous studies that SP1 regulated the levels of UCA1 in GC, we postulated that PA might elevate nuclear protein levels of SP1 to increase the cellular UCA1. We firstly performed nucleus-plasma separation assays to detect the nuclear protein levels of SP1 and discovered 0.1 mM PA increased nuclear protein levels of SP1 (Fig. 3a). To further confirm this result, we performed IF assay with the similar results (Fig. 3b). These results together suggested that PA elevated the nuclear protein levels of SP1 to increase cellular levels of UCA1. Besides, we decreased cellular SP1 via siRNA verified by western blot analysis (Fig. 3c) and found PA-induced upregulation of UCA1 could be significantly inhibited by silence of SP1, which was not found between the BSA treated groups (Fig. 3d). These results, taken together, indicated that PA increased the levels of UCA1 mainly via enhancing the nuclear protein level of SP1.

To stepwise explore the biological effects of SP1 and UCA1 in the process of PA-induced GC metastasis, we then decreased cellular levels of SP1, UCA1 or both via siRNA and then performed migration and invasion assays. Through qRT-PCR we discovered that either knockdown of SP1 or UCA1 could specifically decreased the cellular levels of UCA1 among PA treated groups, besides, the cellular levels of UCA1 were similar among groups treated with siRNA of SP1, UCA1 or both exposed to PA, indicating PA increased cellular UCA1 mainly through SP1 (Fig. 3e). Similarly, knockdown of SP1, UCA1 or both could all significantly inhibit the PA-induced promotion of CG migration and invasion to similar extent (Fig. 3f–i). This phenomenon was not found between BSA treated groups (Fig. 3f–i). Summarily, mainly through SP1/UCA1 axis, PA promoted GC metastasis.

### PA facilitates GC metastasis via FABP5/SP1/UCA1 signaling

**Fig. 4:**
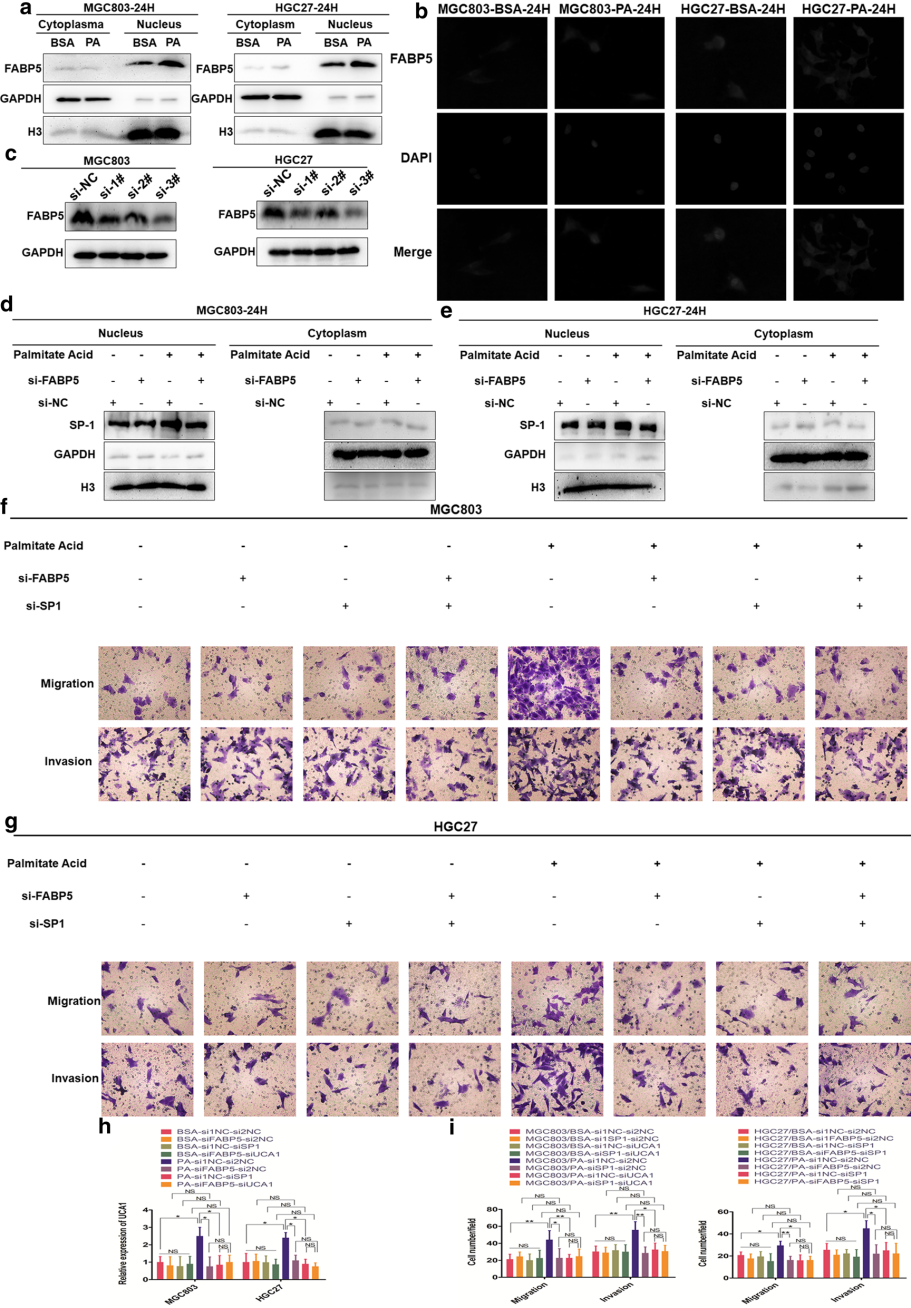
PA facilitates GC metastasis via FABP5/SP1/UCA1 signaling. **a** Western blot analysis of cellular distribution of FABP5 in MGC803 and HGC27 treated with either 2% BSA or 0.1 mM PA for 24 h. **b** IF images represent cellular distribution of FABP5 in MGC803 and HGC27 treated with either 2% BSA or 0.1 mM PA for 24 h. **c** Western blot analysis of the efficiency of siRNA of FABP5. **d**, **e** Western blot analysis of nuclear protein level of SP1 in MGC803 and HGC27 treated with either 2% BSA or 0.1 mM PA for 24 h based on knockdown of FABP5. **f**, **g** The effects of transfection with si-FABP5, si-SP1 or both and their negative controls on GC cells migration and invasion treated with either 2% BSA or 0.1 mM PA (magnification: ×200). **h** qRT-PCR analysis of cellular levels of UCA1 in MGC803 and HGC27 transfected with si-FABP5, si-SP1 or both and their negative controls respectively and then treated with either 2% BSA or 0.1 mM PA for 24 h. **i** Histogram shows the number of migrated and invaded cells (magnification: ×200). Five random fields are selected for statistical analysis. Data are shown as mean ± SD of three independent experiments. *P < 0.05, **P < 0.01, ***P < 0.001, ‘NS’ means not significant

To investigate the potential mechanisms underlying the PA-induced elevation of nuclear levels of SP1, we then explored the correlation between FABP5 and PA as it acted as the FAs carrier. As FABP5 perform its biological effects within nucleus [16], we performed nucleus-plasm separation assay and discovered PA indeed facilitated the nuclear transport of FABP5 (Fig. 4a). Besides, we further proved it through IF assay with similar results (Fig. 4b).

To testify the potential correlation between FABP5 and SP1, we specifically knockdown FABP5 via siRNA verified by western blot (Fig. 4c). The result showed that siRNA-#3 could most efficiently decrease cellular levels of FABP5, therefore, siRNA-#3 was utilized in the following assays. Then we performed western blot to detect the effects of FABP5 on SP1. We discovered knockdown of FABP5 could significantly inhibit PA-induced elevation of nuclear levels of SP1 which were not found between BSA treated groups (Fig. 4d, e), demonstrating that PA elevated nuclear protein levels of SP1 mainly via activation of FABP5.

To further cognize the effects of FABP5 and the relation among FABP5, SP1 and UCA1, we decreased cellular levels of FABP5, SP1 or both to detect their influences on cellular UCA1 which turned out that these groups could all significantly reverse PA-induced increase of cellular UCA1 to similar extent. Among BSA treated groups, however, no such difference was found (Fig. 4h). This phenomenon signified that PA upregulated cellular UCA1 mainly via FABP5/SP1 axis (Fig. 4h). We then performed migration and invasion assays to stepwise testify the biological effects of FABP5 and SP1. Consistently, knockdown of FABP5, SP1 or both could all abolish the tumor-promoting effected induced by PA to similar extent (Fig. 4f, g, i). This result was not found between BSA treated groups (Fig. 4f, g, i). In conclusion, PA facilitated GC metastasis mainly via FABP5/SP1/UCA1 signaling.

## Discussion

GC has a prediction toward omentum which is mainly composed of adipose tissues [9], indicating that FAs may contribute to GC’s peritoneal metastasis. Besides, we previously illustrated that long non-coding RNA UCA1 contributed to the peritoneal metastasis of GC [20]. Therefore, it necessitates the study of the potential connection between FAs and UCA1. FAs include saturated FAs, monounsaturated FAs like OA and polyunsaturated FAs like DHA. Within the area of GC, the effects of monounsaturated FAs [10] and polyunsaturated FAs on the progression of GC have been illustrated [11]. However, there lacks relate researches about the biological effects of saturated FAs on GC. PA, a C16 long-chain saturated FA constituting one of the major parts of human serum, has been reported to contribute to the distal metastasis of oral cancer [23]. Therefore, PA is utilized in our study. In this study, we find 0.1 mM PA promotes GC cells metastasis via elevating cellular levels of UCA1 which overexpressed in GC tissues compared to gastric normal tissues [19, 24]. And its expression levels in GC tissues negatively correlated with GC patients’ survival using TCGA database.

SP1, a transcription factor performing its functions within nucleus, has been reported to contributed to the progression of GC [25]. We previously elucidated that SP1 directly induced UCA1 transcription [19], we then investigated whether PA could promote the nuclear protein levels of SP1. We discovered that PA indeed elevated GC nuclear protein levels of SP1, and PA-induced increase of cellular UCA1 could be entirely reversed by knockdown of SP1, indicating PA increase cellular UCA1 mainly via SP1. To investigate the underlying mechanisms contributing to the PA-induced increase of nuclear levels of SP1, FABP5 was chosen for stepwise analysis as FABP5 acted as the FAs carrier and participated in the progression of multiple cancers [14, 15, 26]. FABP5 acts as a FAs transporter, only particular ligands could activate it and promote its nuclear transport to perform its biological functions [16, 17]. We discovered that PA could promote the nuclear transport of FABP5, and specifically knockdown FABP5 abolished PA-induced elevation of nuclear SP1, which then decreased cellular UCA1. However, whether it is PA itself or its metabolites that activates FABP5 is not clear and need further exploration. Besides, knockdown of either SP1 or FABP5 inhibited PA-induced increase of cellular UCA1, but co-knockdown of SP1 and FABP5 has no additional effects on UCA1. These results manifest PA up-regulates GC cellular UCA1 mainly through FAPB5/SP1 signaling. Furthermore, both migration and invasion assays certify that PA promotes GC metastasis mainly via FABP5/SP1/UCA1 signaling. However, as PA has multiple biological functions and can positively or negatively control other signaling and the overall impact of PA on FABP5/SP1/UCA1 signaling is not straightforward and may vary on different cell types, thus the impact of PA remains to be clarified. Even so, treatments targeting FABP5 or patients’ diets might provide meaningful opportunities for tumor therapeutic strategies.

## Conclusion

**Fig. 5:**
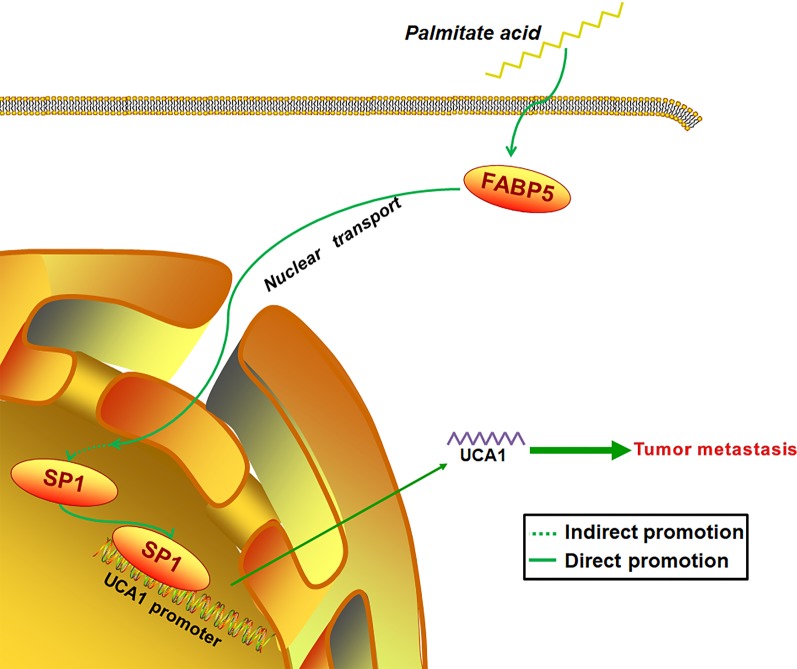
Schematic diagram of molecular mechanisms underlying PA-induced GC metastasis. Schematic diagram shows that PA enters into GC cell, activates FABP5 and promotes its nuclear transport, which then increases GC cell nuclear protein level of SP1. Consequently, the cellular levels of UCA1 is elevated and both the GC cell migratory and invasion properties are improved

In conclusion, the observations suggest the model depicted in Fig. 5, PA enters into GC cell, promotes the nuclear transport of FABP5, which then increase GC nuclear protein levels of SP1. Consequently, the cellular levels of UCA1 is elevated and both the GC cell migratory and invasion properties are improved. Our study connects two hot research areas of tumor, namely, non-coding RNA and FA metabolism together and elucidates a new molecular mechanism underlying the PA induced GC metastasis via FABP5/SP1/UCA1 signaling which contributes to efficient prevention and therapeutic strategies for GC.

## Abbreviations

FA: fatty acid
PA: palmitate acid
BSA: FA-free bovine serum albumin
IF: immunofluorescence
FABP5: FA-binding protein 5
UCA1: urothelial cancer associated 1
